# Influences of Horizontal and Vertical Build Orientations and Post-Fabrication Processes on the Fatigue Behavior of Stainless Steel 316L Produced by Selective Laser Melting

**DOI:** 10.3390/ma12244203

**Published:** 2019-12-14

**Authors:** Paul Wood, Tomasz Libura, Zbigniew L. Kowalewski, Gavin Williams, Ahmad Serjouei

**Affiliations:** 1 Institute for Innovation in Sustainable Engineering, College of Engineering and Technology, University of Derby, DE1 3HD, UK; G.Williams1@derby.ac.uk; 2 Department of Experimental Mechanics, Institute of Fundamental Technological Research of the Polish Academy of Sciences, 02-106 Warsaw, Poland; tlibura@ippt.pan.pl (T.L.); zkowalew@ippt.gov.pl (Z.L.K.); 3 Department of Engineering, School of Science and Technology, Nottingham Trent University, Nottingham, NG11 8NS, UK

**Keywords:** selective laser melting, stainless steel 316L, fatigue, defect, fracture

## Abstract

In this paper, the influences of build orientation and post-fabrication processes, including stress-relief, machining, and shot-peening, on the fatigue behavior of stainless steel (SS) 316L manufactured using selective laser melting (SLM) are studied. It was found that horizontally-built (XY) and machined (M) test pieces, which had not been previously studied in the literature, in both stress-relieved (SR) or non-stress-relieved (NSR) conditions show superior fatigue behavior compared to vertically-built (ZX) and conventionally-manufactured SS 316L. The XY, M, and SR (XY-M-SR) test pieces displayed fatigue behavior similar to the XY-M-NSR test pieces, implying that SR does not have a considerable effect on the fatigue behavior of XY and M test pieces. ZX-M-SR test pieces, due to their considerably lower ductility, exhibited significantly larger scatter and a lower fatigue strength compared to ZX-M-NSR samples. Shot-peening (SP) displayed a positive effect on improving the fatigue behavior of the ZX-NSR test pieces due to a compressive stress of 58 MPa induced on the surface of the test pieces. Fractography of the tensile and fatigue test pieces revealed a deeper understanding of the relationships between the process parameters, microstructure, and mechanical properties for SS 316L produced by laser systems. For example, fish-eye fracture pattern or spherical stair features were not previously observed or explained for cyclically-loaded SLM-printed parts in the literature. This study provides comprehensive insight into the anisotropy of the static and fatigue properties of SLM-printed parts, as well as the pre- and post-fabrication parameters that can be employed to improve the fatigue behavior of steel alloys manufactured using laser systems.

## 1. Introduction

Additive manufacturing (AM) has been recognized over the last three decades as one of the revolutionary manufacturing technologies in various industrial areas, such as military, medical, devices, aerospace, and automobile industries. Compared to conventional manufacturing, AM has shown unbeatable potential, such as design freedom and flexibility in making functional complex parts while saving time and money. AM processes fabricates parts from CAD data through layer-by-layer adding and joining of the material. Selective laser melting (SLM), among AM technologies, has become one of the main fabrication methods for manufacturing parts out of metals in stainless steel, nickel, titanium, and aluminium alloys [1,2,3,4,5,6]. The improper choice of metal AM process parameters, such as the powder material type, power density of the laser, velocity and method of scanning, and thickness of the layer, can result in the formation of defects of a different morphology, number, and size, which influence the mechanical behavior of the material. Spherical porosity, a lack of fusion (LOF) holes of irregular shapes, and cracks are the three well-known types of defects. Bauereiß et al. [7] explained that spherical porosity has a random distribution within the parts, while LOF holes are spread between the layers. Porosity and LOF defects are produced by three main mechanisms:

1. In some AM technologies which use high-density powers, material might be deposited or melted in keyhole mode, which is a melt pool with a deep and narrow shape. Cunningham et al. [8] showed the threshold, morphology, and different stages of keyhole formation through X-ray imaging, and suggested that different combinations of laser power density and scanning velocity might result in the formation of keyholes;
2. Entrapment of gas within the powders might occur while undergoing atomization. Regular spherical gas pores might also be created if the shielding gas (which is utilized in the chamber to avert the melt pool contamination) or alloy vapors are entrapped inside the melt pool. King et al. [9] showed that in uncontrolled keyhole mode melting, unstable keyholes are repeatedly formed and collapsed and this will result in the formation of entrapped vapor inside the deposit;
3. LOF defects can be formed when the melt pool of a newly deposited layer insufficiently penetrates into the substrate layer [10].

Crack formation is related to the thermal history of AM parts. Metal powders, in SLM methods which use laser powers of a high density, undergo fast melting, followed by a fast solidification process. This results in a fast melt pool cooling rate and a very large temperature gradient, which in turn creates a large residual thermal stress. Tang et al. [11] reported a cooling rate of 10^6^ K/s for AlSi10Mg parts fabricated by SLM and showed that the microstructure of the parts was dependent on the amount of heat energy used to produce them. Gu et al. [12] reported a cooling rate of 10^8^ K/s for SLM titanium parts and concluded that the fast melting and cooling experienced by the part during manufacturing results in the formation of martensitic phase with different microstructural geometries. Zhao et al. [13] showed that materials such as nickel-based alloys with a high thermal expansion coefficient and low thermal conductivity, fabricated using laser-based systems, are susceptible to cracking. To reduce crack formation in SLM, pre-heating the substrate is recommended. Besides LOF defects and gas porosity, the resulting composition of alloys, such as intermetallic mixtures and oxides, provides initiation sites for crack formation. For example, Tang and Pistorius [14] observed that in AlSi10Mg alloy SLM parts, widespread large (over micron size) and small (less than micron size) oxides formed due to metal vaporization, and were the crack initiation points. They showed that the sizes of oxides are related to the SLM pre-processing conditions and the fatigue and tensile behavior of the parts are directly affected by the presence of these oxides.

A major current drawback of metal AM is its high surface roughness, which results in stress concentrations and serves as crack initiation sites, thus inhibiting long fatigue lives. The SLM parts are normally milled or polished to reduce their surface roughness. It has been elucidated through experimental works that the surface roughness of AM materials, if reduced, will result in enhancement of their fatigue behavior. Spierings et al. [15] observed that the fatigue strength of polished and machined vertically-built (ZX) SS 316L test pieces was similar, while the ZX and as-built (AB) testpieces (ZX-AB), with large a surface roughness (Ra ≈ 10 μm), exhibited an inferior fatigue strength compared to the polished and machined testpieces. Uhlmann et al. [16] studied the fatigue behavior of stainless steel (SS) 316L ZX test pieces with the three different surface conditions of AB, vibratory-finished, and machined (turned). They showed that test pieces with machined surfaces displayed the best fatigue performance, followed by vibratory-finished pieces, and AB test pieces showed the most inferior fatigue behavior. Riemer et al. [17] showed that the ZX SS 316L test pieces with a hot isostatic pressing (HIP) and machined surface condition have a higher fatigue limit (at a run-out cycle of 2 × 10^6^) compared to ZX, machined (M), and stress-relieved (SR) (ZX-M-SR) test pieces. Leuders et al. [18] observed slightly improved fatigue behavior for the machined (M) ZX SS 316L test pieces in a non-stress-relieved (NSR) condition (ZX-M-NSR) compared to the SR condition (ZX-M-NSR). They also observed that ZX-M test pieces with HIP showed a considerably lower fatigue strength in the range of low cycles and higher stress; whilst for the case of high cycles and lower stress, a slightly improved fatigue strength compared to the AB and SR conditions was observed. Mower and Long [19] observed that horizontally-built (XY) and AB (XY-AB) SS316L test pieces displayed a considerably higher fatigue strength compared to non-heat-treated or HIPed ZX test pieces. They also showed that the fatigue behavior of ZX test pieces in the high cycle and low stress range was improved after applying HIP without increasing the low-cycle and high-stress fatigue life. Zhang et al. [20] studied the influences of build orientation and post-processing treatments (annealing or hot isostatic pressing) on the fatigue and fracture behaviors of SLM-printed SS 316L in the 10^4^–10^6^ cycles region. They attributed the anisotropy observed in the fatigue data to the ratcheting mechanism in which the accumulation of plastic strain takes place under stress-controlled cyclic loading.

**Figure 1:**
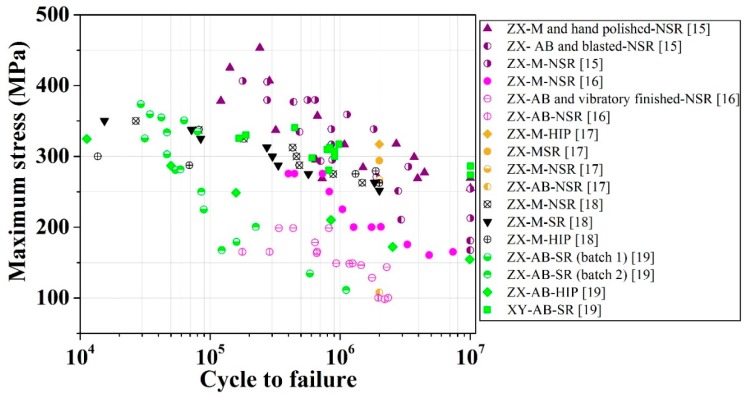
Room-temperature force-controlled stress-life (S-N) data for stainless steel (SS) 316L with different surface conditions (machined (M), as-built (AB), and/or other further post-surface treatments) and heat treatment conditions (stress-relieved (SR), non-stress-relievd (NSR), and Hot Isostatic Pressing (HIPed). The patameters for these tests are as follows: Tension-tension test with R = 0.1, frequency = 50 Hz, and run-out cycle N_f_ = 10^7^ [15]; rotating-bending test with frequency = 100 Hz and run-out cycle N_f_ = 5 × 10^5^ and N_f_ = 2 × 10^6^ [16]; tension-compression test with R = −1, frequency = 40 Hz, and run-out cycle N_f_ = 2 × 10^6^ [17,18]; rotating fatigue test with R = −1, frequency = 25 Hz, and run-out cycle N_f_ = 10^7^ [19].

Aboulkhair et al. [21] argued that, throughout the last decade, the focus of many researches in the area of the laser melting of metals has been on the establishment of functional parts for different applications, as well as the effect of printing parameters on the static mechanical properties. However, the effects of pre- and post-processing parameters, such as build orientation, surface finishing, stress-relief, and/or heat treatment, on the fatigue behaviour of various metals fabricated using laser systems have received comparatively less attention. DebRoy et al. [22] stated that the fatigue analyses of laser-melted metal alloys reported in the literature have been mainly focused on Ti6Al4V and there are, by comparison, only limited works on other metals, such as SS. Furthermore, the literature shows that there are very limited works studying the fatigue life of XY test pieces with different surface and heat treatment conditions applied, and thus a lack of data on the anisotropy of the fatigue properties of SLM parts. The influence of the surface treatment due to the shot-peening (SP) surface on the fatigue behavior of traditionally manufactured material products has been studied. For example, Shiozawa et al. [23] studied the fatigue behavior of high-strength bearing steel in the range of 10^3^ to 10^9^ cycles. They heat treated and shot-peened the parts and observed a sub-surface fracture mode for the tested test pieces in the entire domain of applied stresses. However, the effect of SP on the fatigue behavior of AM parts has not been reported much in the literature. Researchers have studied force-controlled fatigue tests using tension or rotating methods for steels, such as SS 17-4PH [19,24,25], SS 15-5PH [15,16,17,18], and SS 316L [15,16,17,18,19], produced using different laser systems such as EOS M270, Concept Laser M1, etc. There are also limited strain-controlled fatigue analyses of laser-printed steel in the literature [26,27]. Figure 1 shows the stress-life (S-N) data which have been extracted, with a reasonable degree of accuracy, from literature data, for SS 316L with different surface and heat treatment conditions.

This paper describes the influences of build orientation and post-fabrication processes on the fatigue behavior of SS 316L fabricated via the SLM method. Building orientation relates to horizontal (XY) and vertical (ZX) orientations, and post-fabrication processes include those which are stress-relieved (SR) or non-stress-relieved (NSR), as-built (AB), machined (M), and shot-peened (SP). Measurement of the tensile stress–strain behavior of flat test pieces, as well as force-controlled fatigue tests, were conducted in uniaxial tension-tension mode for test pieces of a circular cross-section. The relationship between surface roughness and the fatigue behavior was examined through surface characterization. Metallography was performed using optical microscopy on the undeformed tensile test pieces to examine the microstructures and voids in the material. Fractography of the tensile and fatigue test pieces was performed to investigate the links between the process parameters, microstructure, and mechanical properties for SS 316L and the fatigue–crack initiation sites.

## 2. Materials and Methods

### 2.1. Specimen Manufacturing

**Figure 2:**
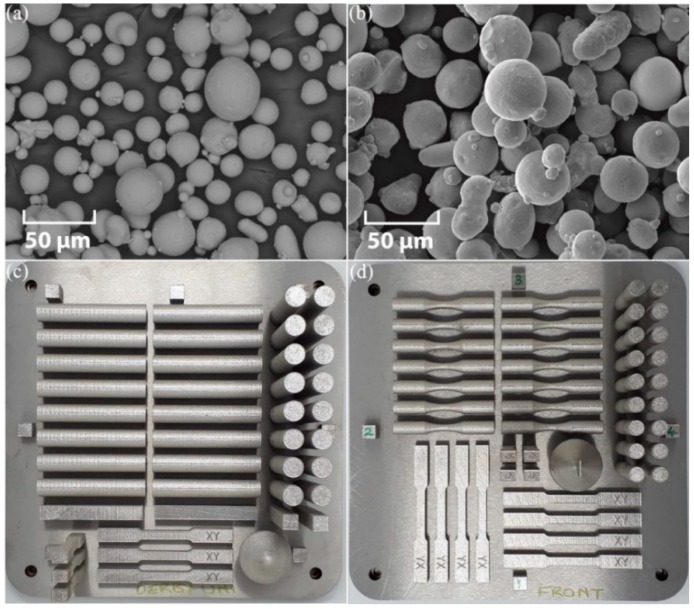
SEM image of SS 316L-0410 (**a**) unused powder and (**b**) mix of unused and used powder; (**c**) stress-relieved (SR) build plate with rods and tensile test pieces; and (**d**) non-stress-relieved (NSR) build plate with fatigue and tensile test pieces.

A Renishaw AM 250 SLM system with a Gaussian beam continuous wave (CW) laser (200 W power, 70 μm spot size, and 1070 nm wavelength) was used to manufacture the SS 316L test pieces. The unused SS 316L-0410 powder was obtained from the supplier, having a particle size in the range from 15 to 45 μm. In this work, a mix of unused and recycled powder was applied for printing. The scanning electron microscopy (SEM) micrographs of the unused powder, as well as a mixture of unused and recycled powder, are shown in Figure 2a,b.

**Figure 3:**
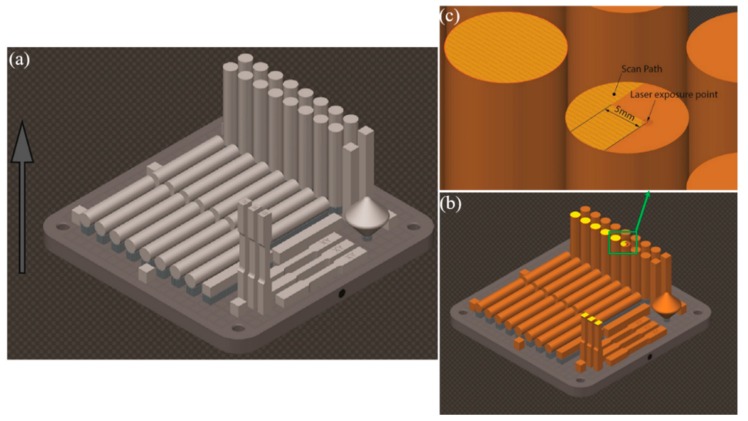
(**a**) Schematic of one of the build plates, in which the arrow shows the building direction; (**b**) build plate half way through printing, in which the highlighted areas show the scanned sections of the current sliced image being printed; (**c**) enlarged view of the stripe scanning strategy at a laser exposure point.

While the powders appear generally spherical, there are some smaller particles and splats attached to large particles. These could be partly due to hardened particles which slammed into full particles while being atomized or printed. A constant supply of argon shielding gas was utilized in the build chamber while printing. A layer thickness of 50 µm and stripe scanning strategy with an exposure time of 50 µs and hatch point distance of 50 µm were used for printing. The stripe scanning strategy is one of the techniques that can be used to process powder within a border region (i.e., fill hatch). This strategy splits the area within the border into a strip size (5 mm used in this study) so that the laser moves across the surface of the powder bed in defined segments rather than one continuous section. Figure 3 shows a schematic of one of the printed build plates, with a detailed view of the stripe scanning strategy.

In total, three builds were performed, of which two were non-stress-relieved (NSR) and one was stress-relieved (SR). One SR and one NSR build are shown in Figure 2c,d, respectively. Seven groups of test pieces were prepared out of these three builds, with combinations of XY, ZX, AB, SR, NSR, M, and SP conditions, namely, XY-M-SR, XY-M-NSR, XY-AB-NSR, ZX-M-SR, ZX-M-NSR, ZX-AB-NSR, and ZX-SP-NSR. Cubes were printed, as shown in Figure 2c,d, at the four remote sides of the build plate for density measurement using Archimedes method to gain an insight into the printed parts produced from a mix of unused and recycled powder. The cubes were lightly grit-blasted after printing with an applied pressure of 5 bar to remove the loose powder from the surface. The average measured density of the cubes was 99.1%. The XY-AB test pieces were built on fine supports. These supports were subsequently removed after the wire-EDM cut from the build plate by using a fine file together with a light grit paper. Evidence of supports was removed such that the modified surface was visually similar to the adjacent as-printed surface.

**Figure 4:**
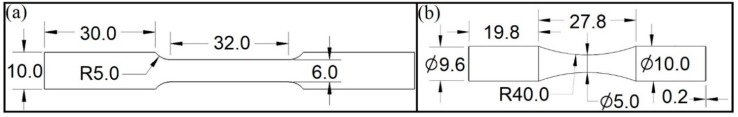
(**a**) Tensile test piece geometry as per ASTM E8/E8M–09 [28] and (**b**) fatigue test piece geometry as per ASTM E466 [29]. Dimensions are in mm.

The tensile test pieces were directly printed as the end-shape as per ASTM Standard E8-09, with the nominal dimensions shown in Figure 4a, in both the XY and ZX direction and with two conditions, namely SR and NSR. The fatigue test pieces were directly printed as the end-shape in the AB condition or machined (M) out of printed rods (of a 12 mm diameter), as per ASTM E466, with the nominal dimensions shown in Figure 4b, in both the horizontal XY and ZX direction. Wrought (W) round bars were machined (M) to the net-shape tensile and fatigue test piece geometries (named W-M). In general, the straightness of the XY and ZX samples in both AB and M conditions was very good, such that the secondary bending effect on fatigue life could be considered negligible.

A Sandvic CoroTurn 107 insert was used for turning of the test pieces which needed machining. First, rough turning was performed using a 300 rev/min spindle speed, 0.1 mm/rev cutting feed rate, and 0.4 mm depth per pass. Finish turning was then performed using parameters of a 600 rev/min spindle speed, 0.1 mm/rev cutting feed rate, and 0.2 mm depth per pass.

### 2.2. Stress-Relieving (SR)

Residual stress which is imposed on the metal AM parts is usually relieved using SR. To study the effect of SR on the tensile and fatigue properties of the test pieces, the SR was performed on one of the builds for 6 h at 470 °C, followed by a period of slowly cooling off, prior to wire-EDM cutting of the test pieces from the build plate.

### 2.3. Chemical Composition Analysis

It is important to check that the chemical composition of the printed test pieces are within the specification of the SS 316L material. The composition elemental analyses of the XY-AB-SR and XY-AB-NSR test pieces were performed using the inductively coupled plasma optical emission spectroscopy (ICP-OES) method.

### 2.4. Shot-Peening (SP)

One group of ZX-AB-NSR fatigue test pieces was surface treated by shot-peening (SP), using fine glass particles of a 250 µm mean diameter, at a pressure of 4 bar for 120 s, applied to each test piece. The test pieces, while being shot-peened, were rotated using a station built for this purpose, to achieve a surface with repeatable precision.

### 2.5. Wide-Angle X-ray Diffraction (WAXD)

ZX-AB-NSR test pieces were shot-peened (SP) and the wide-angle X-ray diffraction (WAXD) method was used on both ZX-AB-NSR (as the reference sample) and ZX-SP-NSR samples to measure the compressive stress applied due to SP. Diffraction patterns for samples were obtained using Bruker D8 Discover equipment with a Cu X-ray source (1.5418Å K-alpha average). The parameters used in the equipment had an angular range (2θ) of 20 to 100 degrees with a step value of 0.02 degrees and counting time per step of 1.25 s. Scans were done using a Bragg–Brentano coupled θ-2θ reflective mode, where the source and one-dimensional linear detector moved simultaneously and symmetrically in regard to the sample placed at the stage. A divergent beam with a 1 mm linear slit was used with 2.5 degree sollers on both primary and secondary beam paths. The positions of the Bragg peaks were determined by the Gaussian fits.

### 2.6. Surface Roughness Characterization

The surface roughness of test pieces was measured using a non-contact profilometer Form Talysurf (Series 2 from Taylor Hobson Precision, UK) system with the following parameters: measurement range of ±0.5 mm, resolution of 0.6 nm, maximum length of the measuring section of 50 mm, and needle radius of 2 μm. The surface roughness was examined within the gauge length area of each test piece to determine the arithmetic mean roughness value, R_a_; the average of distances in the roughness profile between the highest tip and the lowest valley within five sampling lengths gives the roughness depth, R_z_. The measurements were performed at eight positions to ensure reproducibility of the measurement and the average value of these measurements was recorded. Moreover, the standard deviation (STDEV) for each surface roughness measurement was calculated.

### 2.7. Metallography

Metallographic analysis of XY-AB-NSR, XY-AB-SR, ZX-AB-NSR, and ZX-AB-SR tensile test pieces was performed using an Olympus BH2 optical microscope. For this, test pieces were sectioned transverse to the long axis using cold-cutting equipment. The sections were then prepared for microscopic examination by polishing to a 1 μm diamond surface finish using a Bakelite diamond grinding wheel flat of 1200 grit. The samples were then etched with an electrolytic oxalic acid for a duration of 5 to 15 s.

### 2.8. Tensile Testing

A Shimadzu 100 kN servo-hydraulic machine was used for tensile testing at room temperature. A strain rate-controlled speed of 2.5 × 10^−4^ s^−1^ was used in the tests, as per ASTM E8/E8M–09 [28]. Three test pieces of each material with different surface and heat treatment conditions for each print direction were tested.

### 2.9. Fatigue Testing

Force-controlled axial fatigue tests were carried out at room temperature using an MTS 858 fatigue machine with 25 kN load cell and hydraulic grips. The test was performed with a fixed stress ratio, *R* = 0.1, and a 20 Hz sine wave type, to a maximum of a 1 × 10^7^ run-out cycle, except for two tests in the XY-M-SR and ZX-SP-NSR groups, which continued to slightly higher run-out cycles of 1.038 × 10^7^ and of 1.054 × 10^7^, respectively.

### 2.10. SEM Analysis

A LEO 1450VP SEM (Zeiss) scanning electron microscope was utilized to examine the fracture surfaces in order to reveal the microstructural feature effects on the tensile and fatigue behavior of the test pieces. The SEM analysis also helped in detecting the fracture initiation sites, as well as fracture modes, in the tensile and fatigue test pieces.

## 3. Results and Discussion

### 3.1. Chemical Composition, Surface Characterization, and Neutron Diffraction

The results presented in Table 1 alongside the nominal unused SS 316L-0410 powder composition from Renishaw [30] confirm that the test pieces are within the specification of the SS 316L material. The surface roughness measurements for as-built ZX and XY, shot-peened ZX, and machined XY and ZX test pieces are presented in Table 2. The R_a_ and R_z_ values indicate that ZX-AB specimens have the largest surface roughness, followed by XY-AB and shot-peened samples, and machined samples have the lowest surface roughness. The influence of surface roughness on the fatigue resistance of test pieces produced with different surface and heat treatment conditions will be potentially manifested in the S-N diagram, as well as the fracture surfaces examined using SEM.

Based on the analysis of WAXD results, an average value of 58 MPa compressive stress was measured on the surface of the ZX-AB-SP test pieces. The presence of compressive residual stress on the surface of the material hinders the opening of defects and therefore enhances the fatigue resistance of test pieces. This enhancement will be shown in Section 3.4.1, where the S-N diagrams are discussed.

### 3.2. Metallography

**Figure 5:**
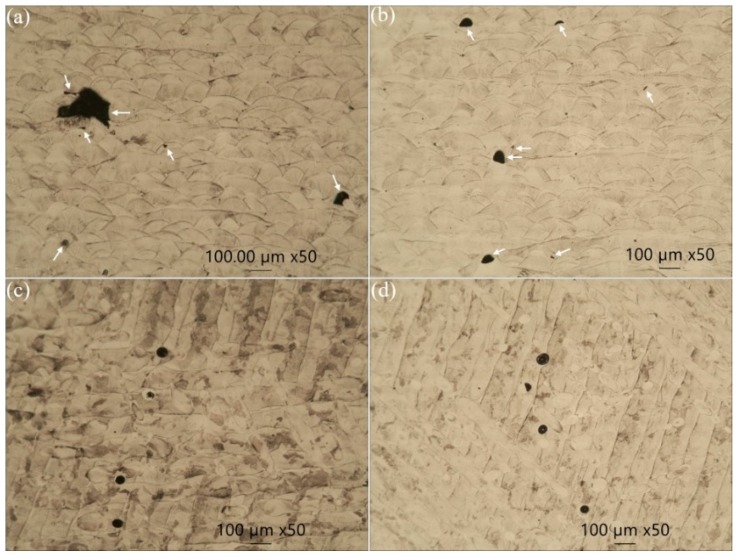
Optical micrographs of defects in: (**a**) XY-AB-NSR, (**b**) XY-AB-SR, (**c**) vertical (ZX)-AB-NSR, and (**d**) ZX-AB-SR tensile test pieces. Arrows in (**a**,**b**) point to lack of fusion (LOF) defects.

The metallographic micrographs are shown in Figure 5. The examined sections of XY and ZX test pieces are perpendicular and parallel with respect to the build plane, respectively. In general, spherical porosity is observed in all the test pieces shown in Figure 5. LOF defects, such as keyhole pores and slit-shaped unmelted regions, as specified by the arrows in Figure 5a,b, are obvious in XY samples. These types of defects, which are formed under the influence of a multitude of pre- and post-process conditions, serve as stress risers and crack initiation points in the test pieces under different loading conditions. The stochastic nature of the LOF defects makes it difficult to precisely foresee the fatigue resistance of parts manufactured using laser systems. Figure 5a,b for XY samples shows apparent melt pools with well-defined boundaries overlapping each other. Parallel laser scanning tracks are obvious in Figure 5c,d for ZX samples.

### 3.3. Tensile Testing

#### 3.3.1. Tensile Mechanical Properties

**Figure 6:**
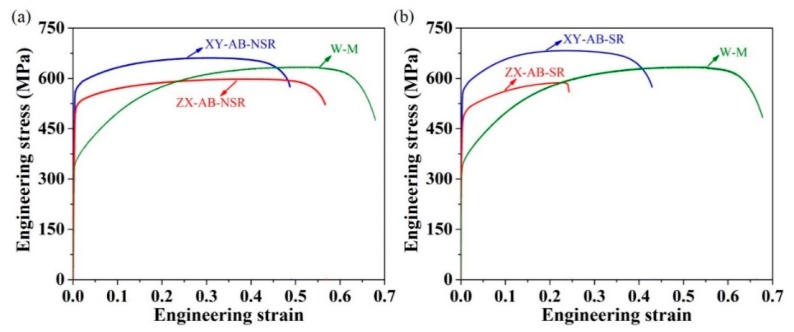
Typical measured room-temperature tensile engineering stress–strain curve for XY-AB and ZX-AB test pieces with (**a**) NSR and (**b**) SR conditions. The data for wrought SS 316L (W-M) is given for reference.

The typical measured room-temperature tensile engineering stress–strain results for XY-AB and ZX-AB test pieces with NSR or SR conditions, as well as wrought SS 316L material (W-M), are shown in Figure 6. The failure strain, yield stress, and ultimate tensile strength (UTS) values are summarized in Table 3. The tensile properties of AB-NSR SS 316L-0410 from Renishaw [30] are also included for reference. Well-defined tensile yielding behavior followed by strain hardening is observed in Figure 6 for all the samples. Strain hardening is followed by necking behavior for XY-AB-NSR, ZX-AB-NSR, and XY-AB-SR test pieces. However, strain hardening for the ZX-AB-SR test piece is continued until failure occurs without necking behavior. XY test pieces in both NSR and SR conditions show a higher yield stress and UTS compared to their ZX counterparts. Considering the maximum values in Table 3 for the UTS, yield stress, and failure strain, applying SR resulted in a reduction of ductility of 13% and 50% for XY and ZX test pieces, respectively. The effect of SR on the yield stress and UTS was negligible (for the yield stress, a slight decrease of 1.4% for the XY test pieces and no change for the ZX test pieces; for the UTS, a slight increase of 3.6% for the XY test pieces and decrease of 1.3% for ZX test pieces).

It has been discussed that the building orientation, due to the part’s aspect ratio, affects the cooling rate and thus anisotropy in both the microstructure and properties [31]. Furthermore, the defects, such as those observed in Figure 5, are sources of stress under axial quasi-static or cyclic loading. For XY samples, the deposited layers and major axes of those defects in Figure 5a,b are parallel to the loading axis, while for ZX test pieces, they are perpendicular to the loading axis. This makes the stress concentration in the ZX samples comparatively more severe than that for the XY samples, and thus more susceptible to defect opening and failure.

Compared to their traditionally manufactured counterparts, as-built AM stainless steels show a generally superior hardness, yield stress, and UTS and inferior ductility. The higher strengths in AM parts are associated with the refined grain structures as a result of fast solidification. Considering the maximum value obtained for the failure strain, yield stress, and UTS, the wrought material (W-M) shows 18.1%, 35.7%, 3.5%, and 107.1% higher ductility compared to XY-AB-NSR, XY-AB-SR, ZX-AB-NSR, and ZX-AB-SR test pieces, respectively; XY-AB-NSR, XY-AB-SR, ZX-AB-NSR, and ZX-AB-SR show 65.4%, 63%, 45.1%, and 45.1% higher yield stress compared to the W-M samples, respectively; and XY-AB-NSR and XY-AB-SR, and ZX-AB-NSR and ZX-AB-SR show comparable (4.4% and 8.1% higher and 5.5% and 6.7% lower, respectively) UTS compared to the W-M samples.

#### 3.3.2. Tensile Fracture

**Figure 7:**
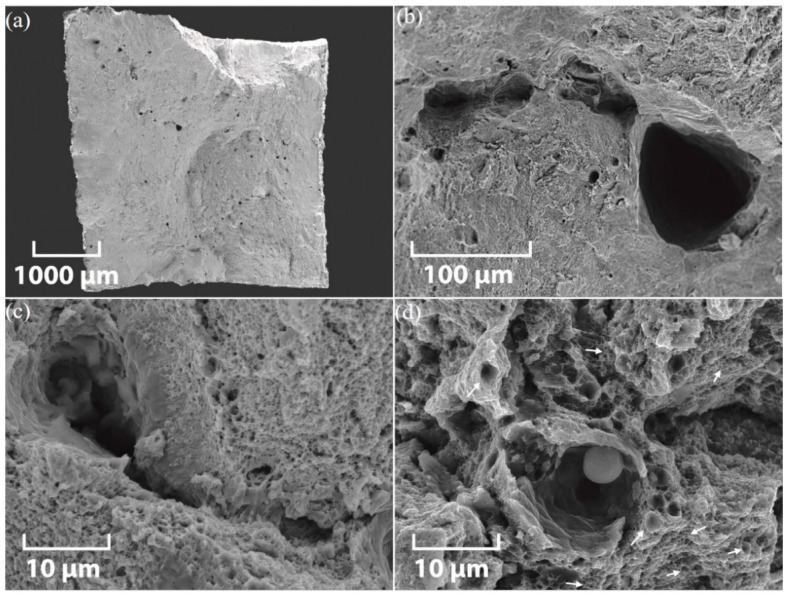
SEM micrographs of the tensile fracture surface of an XY-AB-SR test piece: (**a**) cross-section view and enlarged views of (**b**) pores, (**c**) tear ridges, and (**d**) unmelted powder on the fracture surface. Arrows in (**d**) point to a dimpled fracture.

**Figure 8:**
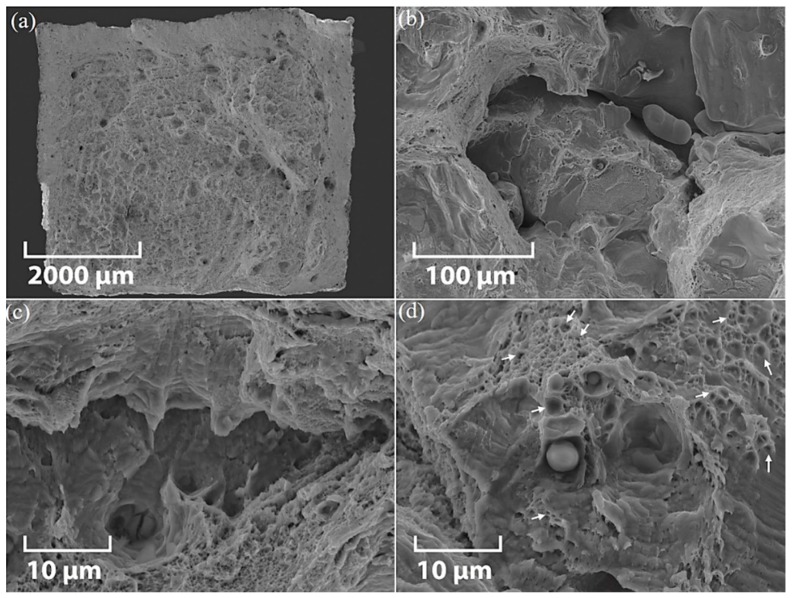
SEM micrographs of the tensile fracture surface of a ZX-AB-SR test piece: (**a**) cross-section view and enlarged views of (**b**) pores, (**c**) a slit-shaped unmelted region, and (**d**) unmelted powder on the fracture surface. Arrows in (**d**) point to a dimpled fracture.

Representative SEM fractographs of the tensile fracture surface of XY-AB-SR and ZX-AB-SR test pieces are shown in Figure 7 and Figure 8, respectively. The overall cross-section view of the tensile fracture surface of the XY-AB-SR test piece is shown in Figure 7a and enlarged views of different types of LOF defects, such as keyhole pores, tear ridges, and unmelted powder, are shown in Figure 7b–d, respectively.

The cross-section view of the tensile fracture surface of the ZX-AB-SR test piece is shown in Figure 8a and enlarged views of different types of LOF defects, such as pores, a slit-shaped unmelted region, and unmelted powder, are shown in Figure 8b–d, respectively. Unmelted powders of the same size shown in Figure 2a,b are observed in Figure 8b,d.

Keyhole pores containing powder are obvious in both fracture surfaces shown in Figure 7 and Figure 8, indicating incomplete melting of the powder particles. These crater-like voids with brittle fracture features are created when the melt pools which are surrounded by unmelted regions are pulled out. The presence of dimples, however, indicates the occurrence of a ductile fracture. The fracture surface of XY-AB-SR test pieces is transgranular, exhibiting ductile corrugations created in the area of cleavage features (Figure 7b,c). The ductile fracture feature occurred when the micron size voids and cracks were amalgamated. The equiaxed dimples can be seen in the tensile fracture surface of XY-AB-SR test pieces shown in Figure 7d. The transgranular surface shown in Figure 8d for the ZX-AB-SR test piece comprises a network of sub-micron to micron-size ductile dimples. These dimples are shallower compared to the deep dimples in XY-AB-SR test pieces shown in Figure 7d. Furuya et al. [32] observed similar types of dimples in the tensile fracture of SS 316L welds. The dimple sizes in their experiments depended on the material production conditions, namely as-received or welded. This implies that microstructural features observed in fracture surfaces highly depend on the process conditions of parts.

### 3.4. Fatigue Testing

#### 3.4.1. S-N Diagrams

**Figure 9:**
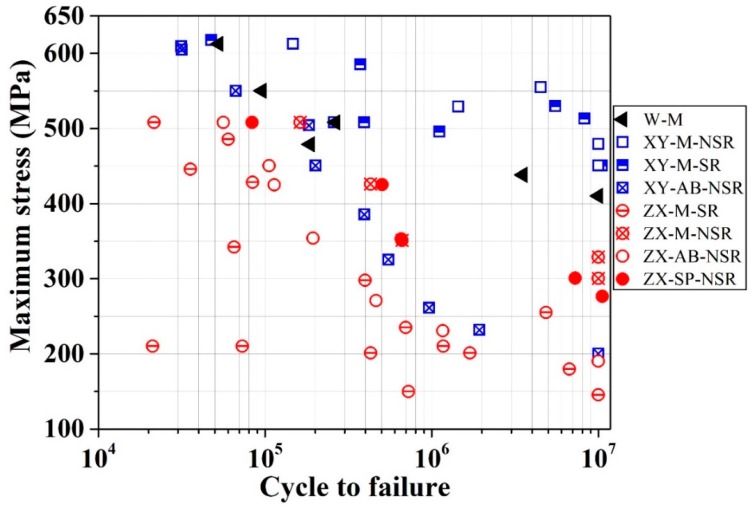
Axial stress-life (S-N) data at a frequency of 20 Hz. The conditions of test pieces are combinations of wrought (W), horizontally-built (XY), vertically-built (ZX), as-built (AB), stress-relieved (SR), non-stress-relieved (NSR), machined (M), and shot-peened (SP) processes.

**Figure 10:**
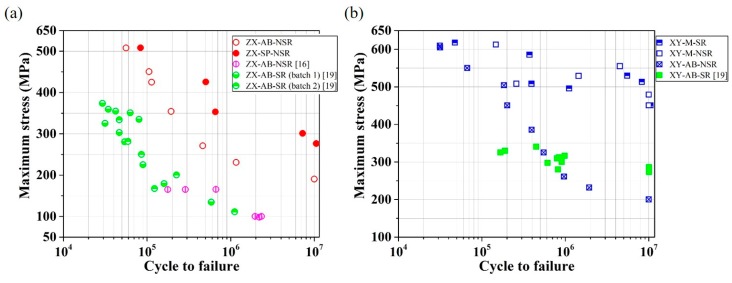
Comparison between current (**a**) ZX and (**b**) XY fatigue data and corresponding data from the literature [16,19], with details shown in Figure 1.

The measured room-temperature stress-life (S-N) values for test pieces with a combination of different conditions, namely W, XY, ZX, AB, SR, NSR, M, and SP, are shown in Figure 9. Comparisons of current ZX and XY fatigue behavior with corresponding data from the literature are presented in Figure 10a,b, respectively. The parameters utilized to produce the parts in this research were not the same as the ones in other works. Therefore, this comparison is not one-to-one and is only included for the sake of showing the trends.

XY-M-SR and XY-M-NSR samples showed the highest fatigue strength among all the studied samples, as seen in Figure 9. Considering the maximum values for the UTS of XY-SR and XY-NSR samples in Table 3, these two samples exhibit fatigue limits (at a run-out cycle of 1 × 10^7^) of around 94% and 98% of their UTS, respectively. They show similar behavior in terms of the fatigue strength and scatter of data, as shown in Figure 9. This shows that SR does not have a considerable effect on the fatigue behavior of horizontally-built (XY) and machined (M) samples. XY-M-SR and XY-M-NSR samples do not show a steep decrease of fatigue strength compared to XY-AB-NSR samples (see Figure 10b). This behavior is similar to the XY-AB-SR data from the work of Mower and Long [19]. Both XY-M-SR and XY-M-NSR samples show a ~10% and 57% higher fatigue endurance limit at 10^7^ cycles compared to the wrought (W-M) and XY-M-SR samples in the work of Mower and Long [19], respectively. XY-M-NSR also show a considerably higher fatigue strength in the range of 2 × 10^5^ to 1 × 10^7^ cycles and a ~125% higher fatigue limit compared to XY-AB-NSR. This confirms the detrimental prevalence of LOF and surface defects, for a high-cycle low-stress regime, which decreases the fatigue strength. Both XY-M-SR and XY-M-NSR display a ~37% and ~136% higher fatigue limit compared to ZX-M-NSR and ZX-AB-NSR, respectively. It is also worth noting that, going from the large-stress and low-cycles region towards the low-stress and high-cycles region, both XY-AB-NSR and ZX-AB-NSR test pieces exhibit a comparatively sharp decline of fatigue resistance. This is largely attributed to the large surface roughness of these samples, which results in the opening of surface defects and immature fatigue fracture.

ZX-M-SR test pieces display a clear increase of fatigue strength with a decline of the stress value similar to the ZX-AB-NSR and XY-AB-NSR samples. This is congruous with the trend seen for the ZX samples in the work of Mower and Long [19], shown in Figure 10a. ZX-M-SR samples display much larger dispersion and a considerably inferior fatigue strength in the range of 2 × 10^5^ to 1 × 10^7^ cycles compared to ZX-M-NSR samples. This can be attributed to the lower ductility of SR samples compered to NSR samples (see Table 3 for a comparison of strain to failure), which means that there is less compensation of stress risers, such as LOF defects. In other words, the sample will be more prone to sudden fracture due to a lower ability to deform plastically.

The comparison of ZX-SP-NSR and ZX-AB-NSR samples (see Figure 10b) reveals the influence of shot-peening in increasing the fatigue strength by ~20% to ~45% in the range of 1 × 10^5^ to 1 × 10^7^ cycles. ZX-SP-NSR samples display a fatigue strength very close to that of ZX-M-NSR. This implies that for the ZX fatigue behavior, the combined effects of compressive stress and a comparatively larger surface roughness (R_a_ = 4.1 μm; see Table 2 for the roughness values) due to shot-peening are comparable to the much lower surface roughness (R_a_ = 0.66 μm) due to machining. ZX-SP-NSR samples also show a considerably higher fatigue strength compared to ZX-AB samples in the works of Mower and Long [19] and Uhlmann et al. [16].

All the fatigue experiments in this work were terminated at a run-out cycle of 1 × 10^7^, with the exception of the two test groups XY-M-SR and ZX-SP-NSR, which were continued to slightly higher run-out cycles of 1.038 × 10^7^ and of 1.054 × 10^7^, respectively. The former test was manually terminated before breakage, while in the latter test, the sample broke. This shows that the S–N curve of SLM-printed samples does not necessarily retain a plateau above the 1 × 10^7^ cycle. Therefore, it would be of significance to study the fatigue behavior of SLM-printed samples in the gigacycle range in the future.

#### 3.4.2. Fatigue Fracture

**Figure 11:**
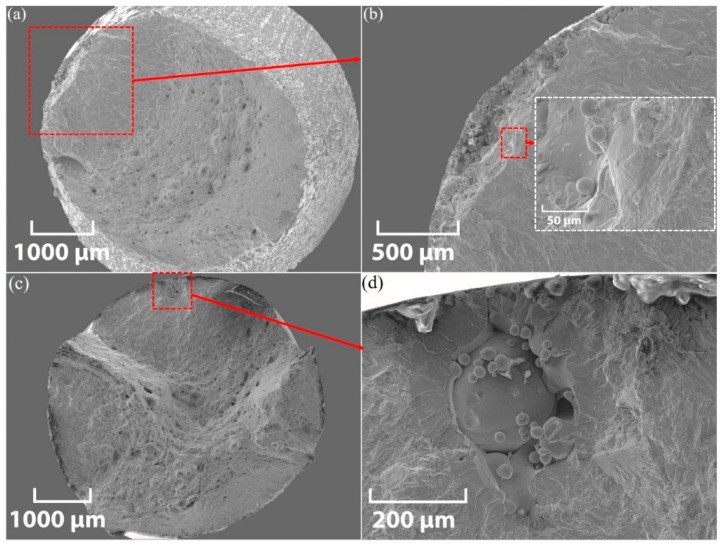
SEM micrograph for fatigue fracture of an XY-AB-NSR test piece (σ_a_ = 550.1 MPa and N_f_ = 66,780): (**a**) cross-section view and (**b**) enlarged view of the crack initiation point; the inset shows more magnified features of the fractured surface with unmelted powder and a ring-shape separation site; SEM micrograph for fatigue fracture of a ZX-M-NSR test piece (σ_a_ = 508.2 MPa and N_f_ = 1.63 × 10^5^): (**c**) cross-section view and (**d**) enlarged view of the fracture initiation site.

Representative SEM cross-section views of an XY-AB-NSR and ZX-M-NSR test piece are shown in Figure 11a,c, respectively. Magnified views of the fracture initiation sites for XY-AB-NSR and ZX-M-NSR test pieces are also shown in Figure 11b,d, respectively. Unmelted powder particles are observed in both fracture surfaces. For the XY-AB-NSR, the fracture initiates from the surface, as shown in Figure 11b, while for ZX-M-NSR, it initiates at the sub-surface (internal fracture), as shown in Figure 11d.

**Figure 12:**
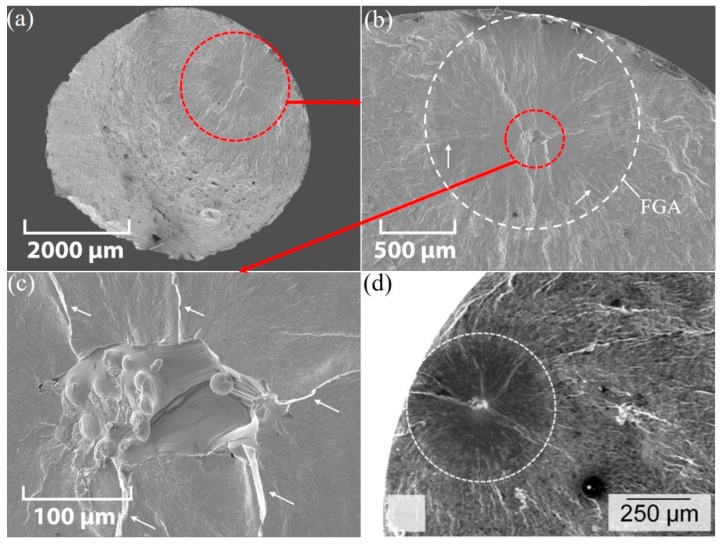
SEM micrograph for fatigue fracture of an XY-M-SR test piece (σ_a_ = 513.2 MPa and N_f_ = 8.25 × 10^6^): (**a**) cross-section view, (**b**) enlarged view of the internal fish-eye pattern, and (**c**) further magnified view of the center of the fish-eye; (**d**) fine granular area (FGA) (σ_a_ = 550 MPa and N_f_ = 1.14 × 10^8^) in fish-eye fatigue fracture of an AISI 4140 steel which was cast and heat-treated [33]. Arrows in (**b**,**c**) show unstable and stable crack propagations, respectively.

A representative SEM cross-section view of an XY-M-SR test piece is shown in Figure 12a. A larger magnification of the internal crack initiation point is shown in Figure 12b,c.

The larger magnification of the internal fracture site in Figure 12b,c shows a specific look recognized as a fish-eye fracture. The fish-eye fracture pattern has an inclusion in its center and a fine-granular facetted region around the inclusion. This type of internal crack initiation at an inclusion site for traditionally manufactured high-strength steels above 10^6^ and 10^7^ fatigue cycles has been observed. For example, Krewerth et al. [34] observed a fish-eye fracture, as shown in Figure 12d, for a steel AISI 4140 which was cast and heat treated. In this type of fatigue fracture, a crack mostly initiates from a single inclusion or cluster of inclusions with an area near the original inclusion. Shiozawa et al. [35] and Sakai et al. [36] noticed this area in their SEM analysis and called it the granular bright faces (GBF) area and fine granular area (FGA), respectively. Li et al. [37] explained the three phenomena occurring while the interior crack is propagated through the strong steels and alloys:

(1) Small crack propagation within the FGA;
(2) Crack stable propagation in the fish-eye region outside of the FGA;
(3) Unstable crack propagation away from the fish-eye.

Similar unstable and stable crack propagations are observed for SLM-printed parts in Figure 12b,c, respectively. While the surface defects promote stress concentration in the surrounding region and may initiate the crack, machining the surface to reduce the surface roughness results in delaying the surface fracture mode. This shift of fracture mode from the surface to sub-surface may be readily explained by comparing the fracture initiation of as-built (AB) and machined (M) test pieces shown in Figure 11 and Figure 12, respectively.

**Figure 13:**
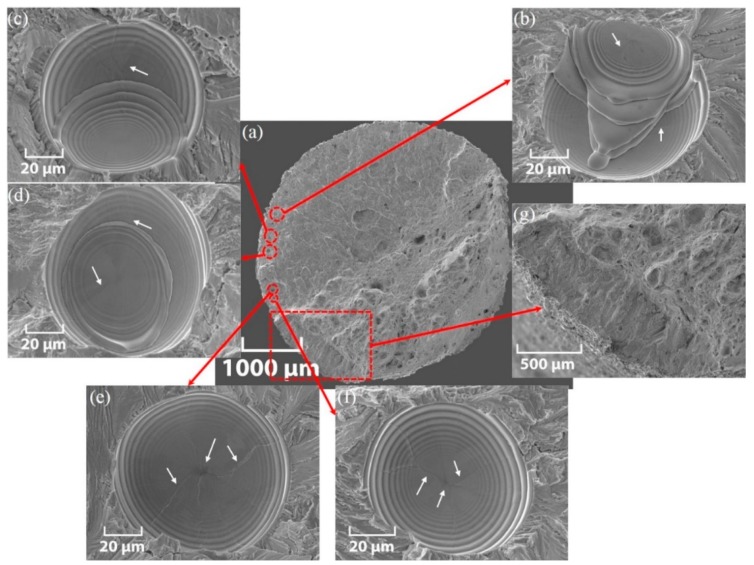
SEM micrograph of the fatigue multi-fracture of a ZX-AB-NSR test piece (σ_a_ = 354 MPa and N_f_ = 1.94 × 10^5^): (**a**) cross-section view, (**b**–**f**) magnified inclusions, and (**g**) enlarged view of a further surface fracture point. White arrows refer to the micro-crack initiation site at the center of the inclusion or cracks propagated from the center of the inclusion.

The SEM cross-section view of a ZX-AB-NSR test piece is shown in Figure 13a. An enlarged view of spherical defects with stair features present throughout the fractured surface is shown in Figure 13b–f. Figure 13g shows the enlarged view of a further surface fracture point. The stair features shown in Figure 13b–f for the fatigue fracture of a ZX-AB-NSR test piece are reminiscent of growth rings observed in the stem of trees used to estimate their age. The difference is that tree growth rings are on the same plane, while those in Figure 13b–f have depth.

These kind of spherical stair defects have not been observed and the reason for their formation has not been explained in the literature. An explanation for the formation of these features is proposed as follows: Powder bed fusion (PBF) technologies like SLM involve spreading and melting of the powder material over previously deposited layers. Flows of melted deposited layers are apparent in Figure 13b. The spherical defects such as those shown in Figure 5c,d for ZX test pieces are present between deposited layers. It is hypothesized that these defects gradually deform and acquire a stair-form shape upon thinning of the deposited layers under axial cyclic loading.

**Figure 14:**
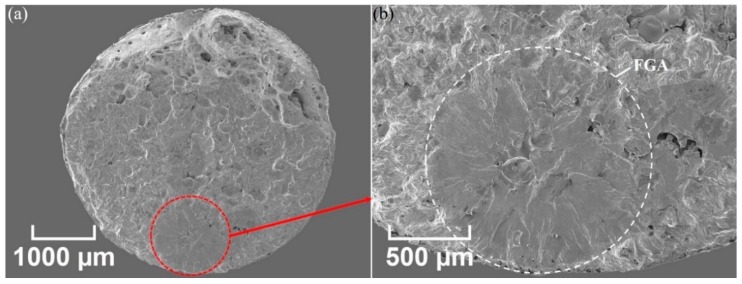
SEM micrograph of the fatigue multi-fracture of a ZX-SP-NSR test piece (σ_a_ = 276.4 MPa and N_f_ = 1.054 × 10^7^): (**a**) cross-section view and (**b**) enlarged view of the internal fish-eye fracture pattern with FGA.

The SEM cross-section view of a ZX-SP-NSR test piece is shown in Figure 14a. A larger magnification of the internal fracture initiation point is shown in Figure 14b. The internal fracture mode of the FGA type shown in Figure 14 was observed for all the SP test pieces in the entire range of stress amplitudes. The influence of the SP treatment, because of the work hardening and presence of compressive residual stress on the surface, shifts the fracture mode from the surface to sub-surface. This is clearly observed when the fracture surfaces of as-built and shot-peened samples shown in Figure 13 and Figure 14, respectively, are compared.

## 4. Summary and Conclusions

In this paper, the influences of build orientation and post-fabrication processes on the fatigue behavior of SS 316L fabricated via the SLM method were studied. Building orientation relates to horizontal (XY) and vertical (ZX) builds, whilst the post-fabrication processes studied include stress-relieved (SR), non-stress-relieved (NSR), as-built (AB), machined (M), and shot-peened (SP) processes. Chemical composition and surface roughness characterizations, metallography, tensile tests, and fractography for both tensile and fatigue samples were performed. The current study draws the following conclusions:

- XY test pieces in both NSR and SR conditions exhibit a higher yield stress and UTS compered to their ZX counterparts. Stress-relief resulted in a reduction in ductility of 13% and 50% for XY and ZX test pieces, respectively; XY-AB-NSR, XY-AB-SR, ZX-AB-NSR, and ZX-AB-SR showed a 65.4%, 63%, 45.1%, and 45.1% higher yield stress compared to the W-M samples, respectively;
- XY-M-SR and XY-M-NSR samples show the highest fatigue strength among all the studied samples. Stress-relief does not have a considerable effect on the fatigue behavior of XY-M samples. Compared to XY-AB-NSR samples, XY-M-SR and XY-M-NSR samples show a considerably higher fatigue strength trend without a steep decrease. This is due to the detrimental prevalence of LOF and surface defects in AB samples resulting in crack initiation and a lower fatigue strength;
- ZX-M-SR samples show much larger scatter and a considerably lower fatigue strength compared to ZX-M-NSR samples. This is attributed to the lower ductility of SR samples compered to NSR samples, which results in more frequent sudden fracture due to a lower ability to deform plastically;
- The comparison of ZX-SP-NSR and ZX-AB-NSR samples showed that shot-peening increased the fatigue strength by ~20% to ~45% in the range of 1 × 10^5^ to 1 × 10^7^ cycles. This can be attributed to the lower surface roughness of SP test pieces compared to AB samples. It was also observed that ZX-SP-NSR samples show a fatigue strength very close to that of ZX-M-NSR. This implies that for the ZX fatigue behavior, the combined effects of the compressive stress and comparatively bigger surface roughness (R_a_ = 4.1 μm) due to shot-peening are comparable to the much lower surface roughness (R_a_ = 0.66 μm) due to machining;
- An internal fish-eye fracture pattern with a surrounding fine granular area (FGA) was detected in the fatigue fracture surfaces of the XY-M-SR and ZX-SP-NSR samples. The shift of fracture mode from the surface to sub-surface was obvious when comparing the fracture initiation of as-built and shot-peened/machined test pieces;
- Stair features were observed in the fatigue fracture of ZX-AB-NSR test pieces. An explanation for the formation of these features is as follows: The spherical defects in ZX test pieces formed between deposited layers during the SLM process. It is hypothesized that these defects are gradually deformed and acquire a stair-form shape upon thinning of the deposited layers under axial cyclic loading;
- Further studies need to be conducted to examine the fatigue behavior of SLM-printed samples in the gigacycle range.

## Figures and Tables

**Table 1:** Chemical composition (mass %) for the horizontal-as-built- non-stress-relieved (XY-AB-NSR) and XY-AB- stress-relieved (SR) test pieces, as well as wrought material obtained from ICP-OES analysis. The chemical composition of the unused SS 316L-0410 powder from Renishaw [30] is also included for reference.

Material/Element	C	Si	Mn	P	S	Cr	Mo	Ni
XY-NSR test piece	0.017	0.65	1.27	0.024	0.007	17.99	2.37	12.59
XY-SR test piece	0.018	0.65	1.27	0.024	0.008	18.05	2.39	12.69
Unused 316L powder	<0.03	<0.75	<2	<0.025	<0.01	17.5–18	2.25–2.5	12.5–13

**Table 2:** Surface roughness measurements of fatigue test pieces using a non-contact system.

Surface Condition	Building Direction	R_a_ (μm)		R_z_ (μm)	
		Value	STDEV	Value	STDEV
As-built (AB)	ZX	21.2	2.3	131.5	5.3
AB	XY	8.5	1.8	51.2	8.9
Shot-peened (SP)	ZX	4.1	0.46	19.7	2.2
Machined (M)	XY/ZX	0.66	0.04	3.7	0.32

**Table 3:** Mechanical properties for as-built (AB) SS 316L in XY and ZX directions with SR or NSR conditions. The tensile properties of AB-NSR SS 316L-0410 are also included for reference.

Process Condition		Failure Strain (%)	Yield Stress ^a^ (MP)	UTS ^b^ (MPa)
W-M ^c^		64.3–67.3	341	637
XY-AB-NSR		54.4–57	542–564	664–665
XY-AB-SR ^d^		47.3–49.6	544–556	675–689
ZX-AB-NSR		56.5–65	451–495	587–602
ZX-AB-SR ^d^		23.6–32.5	467–495	583–594
XY-AB-NSR ^e^		45–47	565–577	680–686
ZX-AB-NSR ^e^		49–59	480–504	585–591

^a^ 0.2% engineering offset yield strength in MPa; ^b^ UTS (ultimate tensile strength) in MPa; ^c^ wrought (W) rod materials machined (M) to the net-shape tensile test pieces; the as-received rods were heat-treated at 1050 °C and then water-cooled; ^d^ stress-relieved (SR) for 6 h at 470 °C and then slowly cooled down in a chamber to room temperature; ^e^ data from Renishaw [30] for as-built (AB), non-stress-relieved (NSR) AM SS 316L-0410.

## Abbreviations

AM	Additive Manufacturing
AB	As-Built
ASTM	American Society for Testing and Materials
CAD	Computer-Aided Design
CW	Continuous Wave
EDM	Electrical Discharge Machining
FGA	Fine Granular Area
GBF	Granular Bright Faces
HIP	Hot Isostatic Pressing
LOF	Lack Of Fusion
M	Machined
NSR	Non-Stress-Relieved
PH	Precipitation Hardening
PBF	Powder Bed Fusion
R	Stress ratio
R_a_	Arithmetic mean roughness value
R_z_	Maximum height of the roughness profile
SEM	Scanning Electron Microscopy
SLM	Selective Laser Melting
SP	Shot-Peening/Shot-Peened
SR	Stress Relief/Stress-Relieved
SS	Stainless Steel
ST	Solution Treatment
STDEV	Standard deviation
UTS	Ultimate Tensile Strength
W	Wrought
WAXD	Wide-Angle X-ray Diffraction
XY	Horizontally built
ZX	Vertically built
σ_a_	Stress amplitude
*N_f_*	Number of cycles to failure or run-out cycle
*2θ*	Diffraction angle
